# Characterization of postural control impairment in women with fibromyalgia

**DOI:** 10.1371/journal.pone.0196575

**Published:** 2018-05-03

**Authors:** Núria Sempere-Rubio, Juan López-Pascual, Marta Aguilar-Rodríguez, Sara Cortés-Amador, Gemma Espí-López, Israel Villarrasa-Sapiña, Pilar Serra-Añó

**Affiliations:** 1 Department of Physiotherapy, University of Valencia, Valencia, Spain; 2 Biomechanics Institute of Valencia, Polytechnic University of Valencia, Valencia, Spain; 3 Department of Physical Education and Sports, University of Valencia, Valencia, Spain; University of Illinois at Urbana-Champaign, UNITED STATES

## Abstract

The main goal of this cross-sectional study was to detect whether women with fibromyalgia syndrome (FMS) have altered postural control and to study the sensory contribution to postural control. We also explored the possibility that self-induced anxiety and lower limb strength may be related to postural control. For this purpose, 129 women within an age range of 40 to 70 years were enrolled. Eighty of the enrolled women had FMS. Postural control variables, such as *Ellipse*, Root mean square (*RMS)* and Sample entropy (*SampEn*), in both directions (i.e. mediolateral and anteroposterior), were calculated under five different conditions. A force plate was used to register the center of pressure shifts. Furthermore, isometric lower limb strength was recorded with a portable dynamometer and normalized by lean body mass. The results showed that women with FMS have impaired postural control compared with healthy people, as they presented a significant increase in *Ellipse* and *RMS* values (p<0.05) and a significant decrease in *SampEn* in both directions (p<0.05). Postural control also worsens with the gradual alteration of sensory inputs in this population (p<0.05). Performing a stressor dual task only impacts *Ellipse* in women with FMS (p>0.05). There were no significant correlations between postural control and lower limb strength (p>0.05). Therefore, women with FMS have impaired postural control that is worse when sensory inputs are altered but is not correlated with their lower limb strength.

## Introduction

Fibromyalgia syndrome (FMS) is a rheumatologic disorder with clinical features such as widespread pain, fatigue, cognitive symptoms and mood disorders, such as depression and anxiety [1–4]. Moreover, previous studies have concluded that people with FMS may also have altered perception or interpretation of audiovestibular inputs due to neural disintegration at brainstem level [5] and some sensory or motor deficits and suboptimal muscle coordination [6] that may affect postural control. Indeed, the study conducted by Bennet et al. (2007) concluded that one of the ten most debilitating symptoms was altered balance, with a prevalence of 45% [7].

So far, several studies have used objective devices to assess postural control in this population [8–11] using different tests that discriminated which of the different sensory inputs that are responsible for postural control (i.e. visual, proprioceptive and vestibular systems) were impaired. All of them explored the impact of FMS on postural control variables in the time-domain, showing a larger area and higher velocity of the center of pressure (COP) [11,12] or an altered postural stability rate in FM patients [8–10,13] compared with healthy population. Nevertheless, although the postural control system is considered a non-linear system, where reactions are not proportional to the applied stimuli [14,15], none of the studies in this population have used non-linear variables to the assessment of the postural control. Conversely, this approach has been used in studies conducted on other populations. Indeed, people with vertigo show a posturographic signal with a small amount of complexity which makes the switch of behavioral modes more difficult and constrain the postural adaptation needed to select sensory information from the surrounding environment [16]. Non-linear variables have also demonstrated more rigid and less adaptable balance in people with Multiple Sclerosis [17] and hypermobility [18] and they have been used as a predictor of the risk of falls [19]. Therefore, this approach could provide complementary information about the ability to maintain the postural control in FM population.

As described above, postural control is possible because of correct linkages between vestibular, visual and somatosensory information [20,21], therefore it is of particular interest to know the contribution of these sensory inputs to the postural control in FM population. Besides, there are other contributing factors that may influence postural control, as can be observed in studies focusing on mood conditions such as anxiety, where high levels of anxiety have been associated with impaired balance control [22,23]. Other studies have focused on the motor effectors instead, suggesting that the ability of people with FMS to maintain balance may also be affected by the loss of strength that they suffer [24]. Similar findings have been shown in other studies on elderly people [25], patients with cystic fibrosis [26] and people with bone injuries [27,28].

Although both signs (i.e. anxiety and loss of strength) are common in people with FMS [2,29–32], so far no previous studies have analyzed the relationship between lower limb strength and the ability to maintain static balance in women with FMS or the impact of anxiety on their static postural control. Understanding this possible relationship could help therapists to design cause-based treatment plans to improve balance, including strength exercises or focusing on other aspects that are responsible for postural control (i.e. proprioception, visual disturbance, anxiety, etc.).

Our main objective was to determine whether women with FMS suffer impaired postural control in comparison with their healthy counterparts and to study the sensory contribution to postural control in this population. The impact of self-induced anxiety on postural control was also analyzed. In addition, we explored the relationship between postural control and lower limb strength.

## Materials and methods

### Participants

The study design was cross-sectional and purposive sampling (specifically, modal instance sampling) was used to select the study participants. The FMS group (FMG) was composed of 80 women between 43 and 70 years of age who had been diagnosed with FMS. They were recruited from several Fibromyalgia associations in Spain over a period of a year and a half. The control group (CG) was composed of 49 age-matched healthy women. The inclusion criterion for the FMG was diagnosis based on 2010 American College of Rheumatology criteria: widespread pain index (WPI) ≥ 7 and symptom severity (SS) scale ≥ 5 or WPI = 3–6 and SS scale score ≥ 9. Additionally, the symptoms had to have lasted for at least 3 months [4]. For both groups, the exclusion criteria comprised an inflammatory rheumatic disease or an inner ear disorder, the use of antidepressant opioid or sedative drugs, current vertigo or dizziness, visual loss, neurological disorder, peripheral neuropathy and surgery within the past year that would cause balance deficits.

The Institutional Review Board (IRB) of the University of Valencia approved all the procedures that were performed in accordance with the principles of the World Medical Association’s Declaration of Helsinki. Written informed consent was obtained from the participants before the tests started.

### Procedures

#### Anthropometric and clinical measurements

Foot-to-foot bioelectrical impedance was measured with the Tanita bc-601 Body Fat Analyzer (Tanita Corp., Tokyo, Japan) [33,34]. Subjects stood on the metal sole plates of the device wearing only their underwear and all measurements were made after a period of 10 min standing in order to minimize potential errors from acute shifts in fluid distribution. Body mass index was estimated for all participants using the standard prediction equations provided by the manufacturer. Furthermore, perceived pain intensity was measured on a 10-cm visual analog scale (VAS) consisting of a continuous line between two endpoints, with 0 being no pain and 10 being maximum tolerable pain.[35].

#### Postural control

The postural control test was performed using the Wii Balance Board (WBB) (Nintendo, Kyoto, Japan) force platform. Previous studies have validated this device as a good means of analyzing postural control in the standing position [36,37]. The platform was placed on a stable surface on the floor to avoid signal distortion and noise. Subjects were asked to place their feet hip-width apart, toes pointing forward and arms relaxed beside their side in all the tests. A reference point was situated 2 metres in front of the subject at eye level. All the subjects were briefed on the importance of maintaining this position and were asked to avoid any body movement. The subjects performed two consecutive 60-second repetitions [38] of five different tests in a random order. They rested for 30 seconds between tests and repetitions, unless they needed extra time. All the tests are described below.

**Standing position with eyes open (*EO*):** in the previously described position, and with their eyes open, they maintained the position for 60 seconds. In this test all sensory inputs (i.e. visual, proprioceptive and vestibular) were intact, so this was used as a control measurement.

**Standing position while recalling a stressful day (Dual task [*DT*]):** the participants had to maintain the bipedal standing position with their eyes open, recalling a stressful day in their life. They were instructed to think about a common stressful day for two minutes before the test started. They subsequently recounted the stressful events to the physiotherapist in the order in which they occurred. The purpose of this test was to explore the effect of self-induced anxiety on postural control.

**Standing position with eyes closed (*EC*):** the participants performed this test in the previously described position, but with their eyes closed. The purpose of the test was to analyze the extent to which proprioceptive and vestibular inputs can make up for a lack of visual information.

**Standing position on a piece of foam with eyes open (*FEO*):** the test was conducted according to the same procedure as EO, but with the participants standing on a piece of foam. The dimensions of the foam were 45 x 27 x 9 cm and the density was 56.7 kg/m^3^. The purpose of this test was to study the contribution of visual and vestibular inputs to postural control when the proprioceptive system was altered intentionally.

**Standing position on a piece of foam with eyes closed (*FEC*):** the test used was the same as above, but the visual information was overridden. The aim of this test was to analyze the action of the vestibular system when visual information was not present and the proprioceptive input was altered intentionally. The vestibular organ is an inertial measuring system that allows us to sense self-motion with respect to the six degrees of freedom in space in the absence of external sensory cues. Since overriding the rest of the sensory information was not possible with this type of assessment, the main contribution of the vestibular system was assessed with this test, as described above [9,32].

#### Lower limb strength

Maximal isometric strength was assessed using a portable dynamometer NedDFM/IBV (Instituto de Biomecánica de Valencia, Valencia, Spain). Specifically, the isometric quadriceps and hamstring strength of both lower limbs were recorded. To measure this, the participants remained seated without any back support, with their upper limbs beside their body and hips and knees flexed at 90°. When quadriceps strength was assessed, the individuals had to try to extend their knee as hard as they could against the evaluator’s resistance without moving their hip, trunk or upper limbs. When hamstring strength was assessed, the participants were required to flex their knee as hard as they could instead. In this case, they were not allowed to move their hip, trunk or upper limb from the original position either. For both measurements the dynamometer was place on the distal portion of the leg and the evaluator was fixed against a wall to resist the participant’s strength and avoid any leg movement. Three repetitions of each measurement were performed consecutively with a 30-second rest between them, and the mean of the three repetitions was calculated. The two measurements (i.e. quadriceps and hamstring strength) were conducted in a counterbalanced order.

### Reliability studies

Before the study began, we investigated the test-retest reliability of the strength assessment procedures described (isometric measurement of quadriceps and hamstring strength) and the BMI measurement that was recorded with the bioelectrical impedance device, since the reliability of these protocols had not been established previously. For this purpose, a convenience sample composed of 15 healthy women (not the individuals in the CG), with a mean (SD) age of 50.86 (4.63) years, came to our lab to be assessed in two different sessions (eight days apart) by two physiotherapists with extensive experience in biomechanical evaluation. In one of the sessions, the participants were assessed by both physiotherapists, whilst in the other, only one of them conducted the assessment.

### Stabilometry data analysis

The ground reaction force was recorded with a Wii Balance Board (WBB) force plate with four uni-axial vertical force transducers at each corner (Nintendo, Kyoto, Japan). The raw data were acquired using WiiLab software (University of Colorado Boulder, Colorado, USA) for Matlab R2007 (Mathworks Inc, Natick, USA).

Center of pressure (COP) displacement signals were filtered digitally by a Butterworth low-pass filter. We used a 10 Hz cut-off frequency to assure that 99% of power spectral density was below this threshold [36]. The first 10 seconds of each test were excluded from the analysis to avoid any interference from delayed stabilization of the recording equipment after the person stepped onto the force plate [39]. Raw data were recorded at a frequency of 40 Hz. Once the previously described steps had been conducted, three posturographic variables were calculated for each of the five tests performed (each test is considered the mean of the two repetitions performed). An explanation and justification of the selected variables is given below.

**Ellipse (*Ellipse)*:** the 95% confidence ellipse area is a measure of the area that COP traverses. It is determined by taking the radius of the major and minor axes and then fitting an ellipse that would include 95% of the points [40]. This variable was used as the main measure of postural stability. The overall size of the ellipse summarizes the amount of overall motion in square millimeters, and the relative orientation of the ellipse is an indication of the degree to which hip and ankle motion are correlated [41].

**Root mean square of the COP distance *(RMS)*:** this variable measures the average absolute displacement around the mean COP and it is considered a measure of error in the balance control system [42]. It is sensitive to alterations in proprioception [43] and when somatosensory feedback for posture is delayed peripherally, the COP drifts, resulting in a larger than normal RMS [44].

**Sample entropy *(SampEn)*:** this variable indicates the regularity of a time series (i.e. COP path) by calculating the probability of it having repeated itself. The calculation of *SampEn* comprised the following steps [45]: (i) computation of the increase in the recorded COP time series according to the suggestions put forward by Govindan et al. [46]; (ii) computation of the *SampEn* values (using the PhysioToolkit-PhysioNetSampEn software) [47]; and (iii) calculation of the input parameters, m and r, using the empirical approach described by Ramdani et al. [45]. This last step was conducted with our own data and the results achieved the value m = 4 and r = 0.35, with N = 2000, where *N* is the number of input data points, *m* is the length of compared runs, and *r* is the tolerance. The information provided by *SampEn* in a specific population has always been related to their healthy counterparts. When comparing specific pathologic populations with healthy samples, lower *SampEn* values have been associated with higher regularity of the time series, which may be related to a poor capability of the neuromuscular system to adapt to perturbations. In contrast, higher values would be indicative of unstable systems that are too sensitive to perturbations [15].

*RMS* and *SampEn* were calculated for anteroposterior (AP) and mediolateral (ML) directions of COP displacement, which have been related to the contribution of ankle and hip movement to postural control, respectively [48]. In this respect, Winter et al. concluded that the complexity of these control mechanisms depends on the posture adopted, which may require combined strategies. However, for the healthy population, in a side-by-side stance, AP balance is more closely related to ankle control, whereas ML balance is related to hip control [48].

### Statistics

Statistical analysis was performed using SPSS software Version 21 (SPSS Inc., Chicago, IL, USA). Standard statistical methods were used to obtain the mean as a measure of central tendency and the standard deviation (SD) as a measure of dispersion. For the inferential analysis, a mixed model MANOVA [group (FMG and CG) and condition (EO, DT, EC, FEO, FEC)] was performed to establish the effects of group and condition on the dependent balance variables (i.e. *Ellipse*, *RMS* and *SampEn*). When the univariate contrasts showed statistically significant main or interaction effects, pairwise comparisons were performed with the Bonferroni correction. Additionally, Spearman correlations between bilateral lower limb strength (average of dominant and non-dominant lower limb strength) and balance variables were performed for the FMG only. An independent Student’s t-test was performed to verify whether the groups were similar in age, body mass index (BMI) and pain at baseline.

The reliability of quadriceps and hamstring strength recorded by the dynamometer and BMI recorded by the bioelectrical impedance assessment was determined using a repeated measures analysis of variance (ANOVA) to calculate the (2,1) intra-class correlation coefficient (ICC)[49]. Within-day and inter-observer reliability were determined by comparing values obtained by two different observers in two repeated assessments several minutes apart. Between-days and intra-observer reliability were determined by comparing the outcomes of two assessments repeated by the same observer, at least eight days apart. A p-value of 0.05 was accepted as the level of significance.

## Results

### Participants

The FMG was composed of 80 women with a mean (SD) age of 53.95 (6.71) years and a BMI of 26.94 (5.85). The CG was composed of 49 women with a mean (SD) age of 54.47 (5.86) years and a BMI of 25.98 (4.88). There were no significant differences between groups in any of these variables (p>0.05). The FMG showed a mean (SD) pain score of 7.70 (2.04), whilst in CG this score was 1.82 (2.44), which means there were significant differences in pain between groups [t (127) = 14.75, p<0.05, r = 0.79].

### Postural control between groups

The multivariate analysis showed that there was a significant interaction between ‘group’ and ‘condition’ [F_(20, 2028)_ = 3.43, p<0.05, η2 = 0.22] and a significant main effect of ‘condition’ [F_(20, 2028)_ = 28.83, p<0.05, η2 = 0.22] and ‘group’ [F_(5, 123)_ = 4.23, p<0.05, η2 = 0.15]. The univariate analysis showed that the groups presented unequal results of *RMS_AP* in the different test performed since a significant factor interaction was achieved [F_(1.87, 410.35)_ = 5.10, p<0.05, η2 = 0.04]. The postural control in both groups varied according to the test performed because a significant main effect of the ‘condition’ factor was obtained for *Ellipse* [F_(1.83, 231.80)_ = 129.28, p<0.05, η2 = 0.50], *RMS_AP* [F_(3.23, 410.35)_ = 130.78, p<0.05, η2 = 0.51], *RMS_ML* [F_(3.80, 377.96)_ = 191.54, p<0.05, η2 = 0.60], *SampEn_Ap* [F_(3.80, 482.44)_ = 145.82, p<0.05, η2 = 0.53] and *SampEn_ML* [F_(3.61, 458.13)_ = 159.37, p<0.05, η2 = 0.56]. Additionally, a significant main effect of the ‘group’ factor was obtained for *Ellipse* [F_(1, 127)_ = 18.82, p<0.05, η2 = 0.13], *RMS_AP* [F_(1, 127)_ = 18.97, p<0.05, η2 = 0.13], *RMS_ML* [F_(1, 127)_ = 14.68, p<0.05, η2 = 0.10], *SampEn_Ap* [F_(1, 127)_ = 16.46, p<0.05, η2 = 0.12] and *SampEn_ML* [F_(1, 127)_ = 16.86, p<0.05, η2 = 0.12] which implies that the groups presented a different postural control regardless of the test performed.

**Fig 1:**
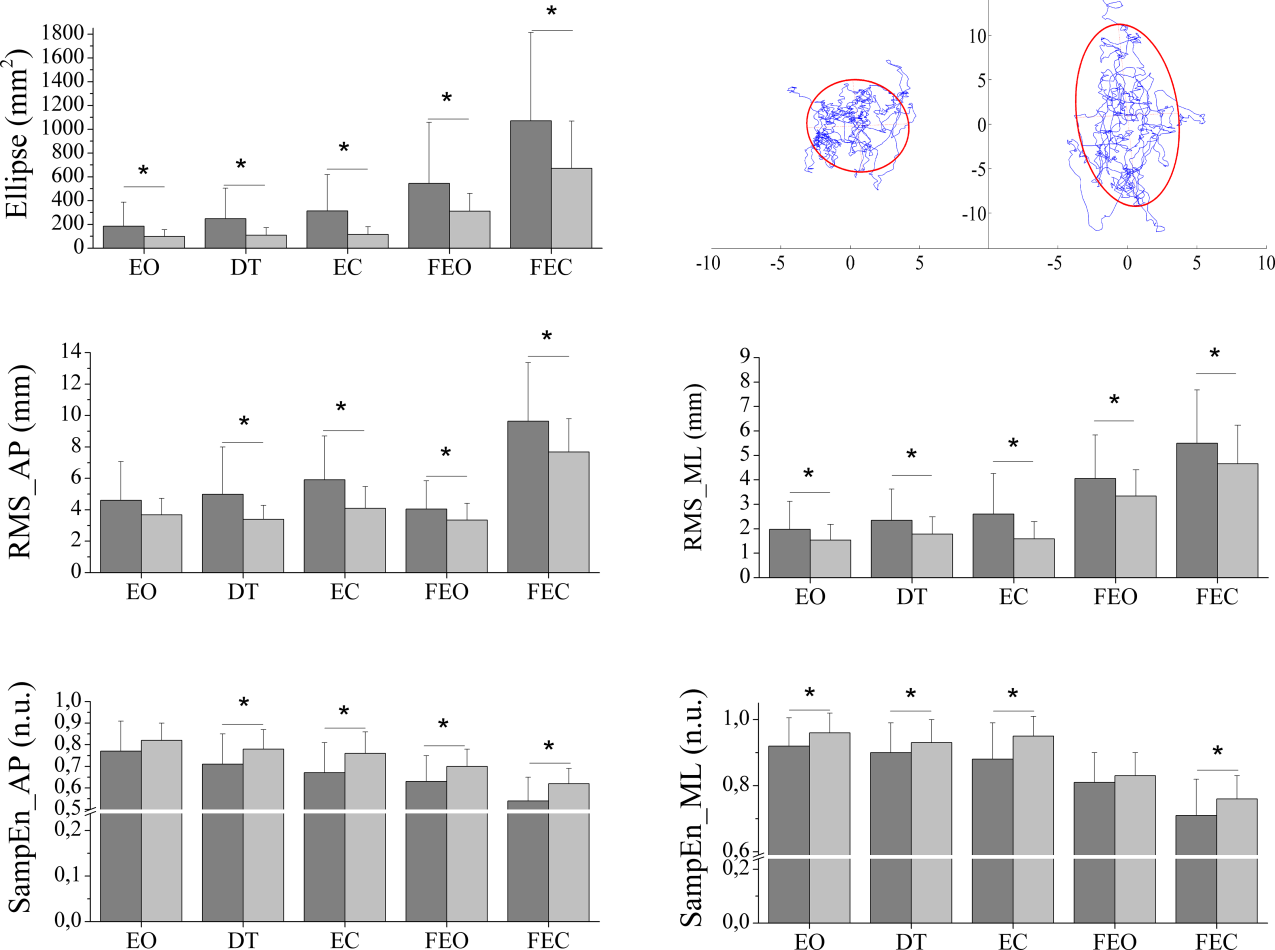
Differences between groups in postural control variables. The bars represent the mean and the error bars, the standard deviation. Dark grey bar = fibromyalgia group; Light grey bar = control group; EO = eyes open test; DT = dual task test; EC = eyes closed test; FEO = foam eyes open test; FEC = foam eyes closed test; AP = anteroposterior; ML = mediolateral; RMS = root mean square of the center of pressure distance; SampEn = sample entropy. At the top right of the panel, the Ellipse of a representative case of the fibromyalgia group (right side) and one of control group (left side) is shown. * indicates significant differences between groups (p < 0.01).

The subsequent between-group analysis showed that there were significant differences in all variables in all the tests conducted, with the exception of *RMS_AP* and *SampEn_AP* for the EO test and *SampEn_ML* for the FEO test. The *Ellipse* and *RMS* values (in both directions) were significantly higher in the FMG than in the CG. Regarding variability, *SampEn* was lower in the FMG than in the CG, which implies a more repetitive movement pattern. Fig 1 shows the between-group results in each of the tests conducted.

### Sensory input results

The posturographic variables were altered in both groups when the difficulty of the task was increased placing a foam on the force plate (i.e. FEO and FEC). This alteration is reflected as an increase in the values of *Ellipse* and *RMS* and a decrease in the values of *SampEn*, compared with the results obtained in those test in which the foam was not used.

Nevertheless, the postural control of the groups was different when the proprioceptive system was not disturbed (i.e. EO and EC). If we compare EC with EO, the CG did not show any significant differences in the postural control variables, except for *SampEn* in the AP direction. However, the FMG showed a significant increase in *Ellipse* and *RMS* in both directions and a decrease in *SampEn* also in both directions. All the data are shown in Table 1.

**Table 1:** Pairwise comparisons of the posturographic variables.

	Ellipse (mm^2^)		RMS_AP (mm)		RMS_ML (mm)		SampEn_AP (n.u.)		SampEn_ML(n.u.)	
	CG	FMG	CG	FMG	CG	FMG	CG	FMG	CG	FMG
**EO**	99.87 (56.23)	184.28 (202.90)	3.68 (1.05)	4.61 (2.46)	1.54 (0.64)	1.98 (1.14)	0.82 (0.08)	0.77 (0.14)	0.96 (0.06)	0.92 (0.09)
**DT**	109.17 (63.64)	248.16 (257.37)^1^	3.40 (0.88)	4.99 (3.00)	1.77 (0.71)	2.34 (1.28)	0.78 (0.09)	0.71 (0.11)	0.93 (0.07)	0.90 (0.09)
**EC**	116.08 (65.39)	313.07 (304.97)^1^	4.10 (1.38)	5.91 (2.79)^1^	1.59 (0.70)	2.60 (1.66)^12^	0.76 (0.10)^1^	0.67 (0.14)^1^	0.95 (0.06)	0.88 (0.11)^1^
**FEO**	311.22 (149.38)^123^	544.07 (515.15)^123^	5.05 (1.30)^123^	6.61 (3.11)^123^	3.33 (1.07)^123^	4.05 (1.79)^123^	0.70 (0.08)^123^	0.63 (0.12)^123^	0.83 (0.07)^123^	0.81 (0.09)^123^
**FEC**	670.95 (397.76)^1234^	1071.85 (741.42)^1234^	7.68 (2.12)^1234^	9.64 (3.73)^1234^	4.66 (1.58)^1234^	5.50 (2.17)^1234^	0.62 (0.07)^1234^	0.54 (0.11)^1234^	0.76 (0.07)^1234^	0.71 (0.11)^1234^

Data are expressed as mean (SD). EO: eyes open; DT: dual task; EC: eyes closed; FEO: foam eyes open; FEC: foam eyes closed; RMS: root mean square variable; SampEn: sample entropy; n.u.: no units. AP: anteroposterior direction; ML: mediolateral direction. Superscript 1, 2, 3 and 4: significant differences versus test 1, 2, 3 and 4, respectively.

### Impact of self-induced anxiety on postural control

When DT and EO were performed, no significant differences were obtained for any of the posturographic variables in the CG (p>0.05). However, the FMG showed significantly higher *Ellipse* values (Table 1).

### Relationship between lower limb strength and postural control

There was no significant correlation between lower limb strength (i.e. quadriceps and hamstring) and posturographic variables (p>0.05).

### Test-retest reliability of strength and BMI assessment procedures

The quadriceps and hamstring strength procedures showed good reliability (2,1 ICC) [49] for dominant lower limb strength, ranging from 0.78 to 0.86. The intra-observer and between-days ICC were 0.83 and 0.78 for quadriceps and hamstring strength, respectively. The inter-observer and within-day ICC were 0.86 and 0.83 for quadriceps and hamstring strength, respectively. Regarding the BMI measurement, the reliability was very good. The intra-observer and between-days ICC was 0.99. The inter-observer and within-day ICC was 1.00.

## Discussion

The results derived from this study show that women with FMS have impaired postural control compared with their healthy counterparts. These results are consistent with previous studies [9–11,13,24] but also yield some new findings about the postural control strategy. In general, there is a significant increase in the values of the linear variables (i.e. *Ellipse* and *RMS*) and a significant decrease in *SampEn* (a non-linear variable). Furthermore, when a sensory input is removed or disturbed, impairment of postural control increases.

Specifically, the *Ellipse* values were an average of 2.03 times higher in women with FMS than in healthy women. This result means that the COP trajectory covers a larger area in this population while standing and it is consistent with the results of a previous study conducted in women with fibromyalgia that reported a similar increase in the area in a bipedal test with eyes open [11]. It also coincides with the results of previous studies conducted in people with Multiple Sclerosis [17] or vestibular system problems [50]. In fact, a previous study concluded that there is a tendency for the linear values of COP, sway area, range of the COP and RMS in both directions (AP and ML) to increase, irrespective of the disability studied [51]. In the case of the population with FMS, a chronic painful condition, it is possible that the motor pattern adapts to this condition and therefore modifies movement and stiffness to protect against further pain. Previous studies in which pain was experimentally induced have reported a larger area. This is attributed to the impact of pain on γ motor neuron activity [52], which could modulate the neuromuscular response. Regarding *RMS*, there is a significant increase in the FMG compared with the CG in both directions, ML and AP. This increase observed in women with FMS may be the result of slowed somatosensory feedback, which may reflect a deficit in the somatosensory feedback loop [43,44]. Nevertheless, a previous study pointed out that an increase in linear values does not always suggest a lack of balance, but may reflect a strategy of skillful individuals to explore their support base by being more flexible [51]. For this reason, in order to confirm the existence of a postural control deficit, *SampEn* was used as a non-linear variability variable. This variable reflects the automatism of postural control and net motor control signal output and comprises the whole body center of gravity and the muscles responsible for postural maintenance [53]. Our results showed that *SampEn* values were generally lower in women with FMS than in healthy women. This decrease in the intrinsic complexity of the steady-state dynamics is associated with a functional decline of the postural control system [54]. Indeed, it is known that a small amount of complexity makes it more difficult to switch behavioral modes, affecting the postural adaptation that is needed to select sensory information from the surrounding environment [55] and indicating that postural behavior is more rigid, which results in a loss of adaptability and local stability [56]. These results are in line with previous studies in which other pathologies were studied, all of which showed lower SampEn or Approximate Entropy compared with healthy people [16,17,51].

In order to further analyze sensory input alterations, as explained in the methods section, different tests were conducted (i.e. EO, EC, FEO and FEC) in which some of the sensory inputs were altered intentionally. The results showed that postural control was worse in both groups (i.e. *Ellipse* and *RMS* values increased and *SampEn* decreased) when the proprioceptive information was altered using a piece of foam (i.e. FEO and FEC). This is justified by the aforementioned linkage between the three sensory inputs. In this case, postural control needs to be maintained even when the proprioceptive information is disturbed, making it more difficult to maintain stability. These results are also in line with those obtained in several previous studies in which increasingly complex tasks were assessed in different populations [16,17,57] and an increase in linear variables and decrease in COP variability were observed.

When EO and EC were compared, the postural control was different depending on which group they belonged to. There were no significant differences between EO and EC in the CG, except for *SampEn_AP*. This may be due to the fact that other sensory input information can make up for the lack of vision [51], although there was a reduction in the complexity of the COP trajectory in the AP direction. However, the FMG could not make up for that information properly and therefore experienced an increase in *Ellipse* and *RMS* and a decrease in variability. It has been suggested that visual input is the most reliable source of information needed for the central nervous system to send the motor response peripherally [58]. Under normal circumstances, when vision is not present (as is the case in EC), the somatosensory information needs to supply this lack of information, but as mentioned above, in women with FMS the somatosensory information seems to be slowed down, as shown in the results, where an increase in *RMS* values was obtained.

The use of a DT to assess the impact of self-induced anxiety on postural control was based on the previously described linkages between balance and anxiety [23]. The role of the parabrachial nucleus (PBN) in conditioning fear and anxiety responses has been demonstrated [59], as have the projections sent from the PBN to the vestibular nuclei [60]. Likewise, the locus coeruleus has been implicated both as an initiator of anxiety responses [59] and as a modulator of vestibular function [61]. Our results showed that the postural behavior in this DT compared with the EO test is different depending on which group the individual belongs to. The CG did not achieve significant differences in any of the posturographic variables, while the FMG presented an increase in *Ellipse* values in the DT compared with the EO test. These results suggest that the DT conducted increases their level of anxiety and vestibular activity is therefore modified and has an impact on postural control. Nevertheless, no significant differences were obtained for *RMS* or *SampEn*, which may be explained by two assumptions. First of all, the test conducted induces the anxiety response by recalling stressor events, but not using any other sensory cues. The use of more information that elicits a greater anxiety response may alter not only the *Ellipse* values but also the complexity of the signal, since it has been demonstrated that some units in the caudal PBN also receive input from eye movement, for example. The other reason that could explain this result is that an increase in linear values does not always suggest a lack of balance, but a more flexible strategy [51]. However, both explanations need to be contrasted with more specific studies. These results therefore suggest that the alteration in postural control is partially modulated by remembrance-related anxiety.

Apart from studying sensory inputs, we explored the possible association between postural control and lower limb strength. Past studies have shown an association between altered postural control and a lack of strength in elderly people and in people with cystic fibrosis [25,26]. In fact, previous studies have suggested that people with FMS may be more prone to fall because they have similar lower body strength to older healthy women [62] and the rate of torque development for hip extension is considered a strong predictor of falls [29]. Nevertheless, the authors of these studies did not find a relationship between falls and peak torque. The results of our correlation analysis between lower limb strength and postural control variables are consistent with the aforementioned results, since we did not find a significant correlation between them either. These results, in conjunction with those previously reported, reinforce the hypothesis of a disrupted somatosensory system in this population as the main cause of their altered postural control. However, future studies should aim to provide further knowledge about proprioceptive information in these people using specific assessment tools. Furthermore, the results of this study should be taken cautiously because due to the fatigue, one of the most common symptoms of this population [4], we only conducted two trials of each condition in the postural control assessment. The number of trials should be expanded when the population assessed could perform the protocol without fatigue in order to improve the reliability of the tests.

## Conclusions

The results obtained from this study demonstrate that women with FMS have impaired postural control compared with their healthy counterparts. Furthermore, their somatosensory system seems to be affected, as shown by the increase in *RMS* and *Ellipse* and reduction in *SampEn* values when visual information is missing. Postural control is partially influenced by self-induced anxiety and it is not related to lower limb strength.

## Supporting information

S1 Dataset Study database. (XLSX)

S2 Dataset Reliability database. (XLSX)
